# Baseline CD4^+^ T Cell Counts Correlates with HIV-1 Synonymous Rate in HLA-B*5701 Subjects with Different Risk of Disease Progression

**DOI:** 10.1371/journal.pcbi.1003830

**Published:** 2014-09-04

**Authors:** Melissa M. Norström, Nazle M. Veras, Wei Huang, Mattia C. F. Proper, Jennifer Cook, Wendy Hartogensis, Frederick M. Hecht, Annika C. Karlsoon, Marco Salemi

**Affiliations:** 1 Division of Clinical Microbiology & Center for HIV Research, Department of Laboratory Medicine, Karolinska Institutet, Stockholm, Sweden; 2 Department of Pathology, Immunology and Laboratory Medicine & Emerging Pathogens Institute, University of Florida, Gainesville, Florida, United States of America; 3 Monogram Biosciences Inc., South San Francisco, California, United States of America; 4 Centre for Health Informatics, Institute of Population Health, University of Manchester, Manchester, United Kingdom; 5 UCSF Positive Health Program, San Francisco General Hospital, University of California, San Francisco, San Francisco, California, United States of America; University of California San Diego, United States of America

## Abstract

HLA-B*5701 is the host factor most strongly associated with slow HIV-1 disease progression, although risk of progression may vary among patients carrying this allele. The interplay between HIV-1 evolutionary rate variation and risk of progression to AIDS in HLA-B*5701 subjects was studied using longitudinal viral sequences from high-risk progressors (HRPs) and low-risk progressors (LRPs). Posterior distributions of HIV-1 genealogies assuming a Bayesian relaxed molecular clock were used to estimate the absolute rates of nonsynonymous and synonymous substitutions for different set of branches. Rates of viral evolution, as well as *in vitro* viral replication capacity assessed using a novel phenotypic assay, were correlated with various clinical parameters. HIV-1 synonymous substitution rates were significantly lower in LRPs than HRPs, especially for sets of internal branches. The viral population infecting LRPs was also characterized by a slower increase in synonymous divergence over time. This pattern did not correlate to differences in viral fitness, as measured by *in vitro* replication capacity, nor could be explained by differences among subjects in T cell activation or selection pressure. Interestingly, a significant inverse correlation was found between baseline CD4^+^ T cell counts and mean HIV-1 synonymous rate (which is proportional to the viral replication rate) along branches representing viral lineages successfully propagating through time up to the last sampled time point. The observed lower replication rate in HLA-B*5701 subjects with higher baseline CD4^+^ T cell counts provides a potential model to explain differences in risk of disease progression among individuals carrying this allele.

## Author Summary

The clinical course of HIV-1 infection is characterized by considerable variability in the rate of progression to acquired immunodeficiency syndrome (AIDS) among patients with different genetic background. The human leukocyte antigen (HLA) B*5701 is the host factor most strongly associated with slow HIV-1 disease progression. However, the risk of progression to AIDS also varies among patients carrying this specific allele. To gain a better understanding of the interplay between HIV-1 evolutionary rate variation and risk of disease progression, we followed untreated HLA-B*5701 subjects from early infection up to the onset of AIDS. The analysis of longitudinal viral sequences with advanced computational biology techniques based on coalescent Bayesian methods showed a highly significant association between lower synonymous substitution rates and higher baseline CD4^+^ T cell counts in HLA-B*5701 subjects. The finding provides a potential model to explain differences in risk of disease progression among individuals carrying this allele and might have translational impact on clinical practice, since synonymous rates, which are proportional to *in vivo* viral replication rates, could be used as a novel evolutionary marker of disease progression.

## Introduction

The clinical course of HIV-1 infection is characterized by considerable variability in the rate of disease progression among patients with different genetic background [1]–[3]. It has been shown that the likelihood of progressing to AIDS for subjects with baseline viral load (VL) around or lower than 10,000 copies/mL is dependent on baseline CD4^+^ T cell counts [4]. Subjects with baseline CD4^+^ T cell counts <750 cells/mm^3^ are at significantly higher risk for progression to AIDS (high-risk progressors, HRPs) than those with CD4^+^ T cell counts >750 cells/mm^3^ (low-risk progressors, LRPs). There is also evidence that HIV-1 genome controls virulence; however, the mechanisms underlying differential risk of progression to AIDS are not fully understood and likely involve both viral dynamics and host immune system [5].

CD8^+^ T cell responses play an important protective role in HIV-1 infection. HIV-1 replication *in vivo* is temporally associated with the appearance of CD8^+^ T lymphocyte responses [6], and the rate of disease progression is dependent on human leukocyte antigen (HLA) class I alleles [7], [8]. HLA-B*5701 is the host factor most strongly associated with slow HIV-1 disease progression [1], [9] and, in subjects with this allele, the CD8^+^ T cell responses target several epitopes in the *gag* p24 gene [10]–[12]. This often results in the evolution of viral variants that escape CD8^+^ T cell responses [13], [14], although there is evidence that some escape mutations in HLA-B*5701-restricted epitopes in p24 might occur at the expense of viral fitness [15]–[17].

HLA-B*5701 subjects with detectable viral load are ideal patients to investigate how the interaction between on-going viral intra-host evolution and immune system relates to risk of disease progression. It has recently been shown that HLA-B*5701 LRP subjects have a larger fraction of polyfunctional cells – i.e. cells producing two or more immune mediators (such as gamma interferon, interleukin-2, macrophage inflammatory protein 1 β, and Perforin) in response to specific HLA-B*5701-restricted epitopes in p24 – than HRPs [5]. At the same time, the study found that HIV-1 evolutionary rate is lower in LRPs compared to HRPs [5]. However, the exact mechanism, evolutionary meaning and clinical implications of these observations are still unclear. The rate of evolution estimated by molecular clock analysis of longitudinally sampled viral sequences is a compounded parameter, which depends on different factors, such as viral mutation (error) rate per generation, generation time (*i.e.* the viral replication rate), as well as the interplay between neutral genetic drift and positive or purifying selection.

In molecular adaptation studies, investigating the ratio of nonsynonymous and synonymous substitutions (*dN/dS*) has often proved to be useful [18], although evaluating the absolute nonsynonymous and synonymous substitutions rates separately can sometimes provide greater insights [19]–[21]. In HIV-1 intra-host evolution, for example, differences in synonymous substitution rates may reflect differences in mutation rate or generation time (i.e. viral replication rate), while different nonsynonymous rates may be linked to changes in selective pressure and effective population size [21]. Lemey *et al*. (2007) showed that HIV-1 disease progression seems to be predicted by synonymous substitution rates, which are indicative of the underlying viral replication dynamics [20]. By using a different method, Lee *et al*. (2008) also showed that the rate of intra-host HIV-1 evolution was not constant, but rather slowed down at a rate correlated with the rate of CD4^+^ T cell decline [19]. However, these studies were performed on patients of unknown HLA type, which makes it difficult to assess the potential impact of the host immune response on viral evolution and disease progression. The mechanism relating evolutionary rates and disease progression may also involve factors such as replication capacity [16] of the infecting virus or T cell activation [22]–[25]. Moreover, virus generation times and the ability of the viral strains to replicate in different environments could be affected by the virus population dynamics in latently infected cells [26], [27].

The present work focuses on a cohort of six untreated HIV-1 infected subjects, all carrying the HLA-B*5701 allele, followed longitudinally from early infection up to seven years. Bayesian molecular clock estimates, based HIV-1 *gag* p24 sequences, were analyzed in combination with *in vitro* viral replication capacity and immune activation data. The integration of experimental data with coalescent-based estimates allowed to develop, for the first time, a possible explanation for the correlation between HIV-1 *in vivo* replication rate and different risk of disease progression in HLA-B*5701 subjects.

## Results

### Analysis of HIV-1 substitution rates in HLA-B*5701 subjects

Analyses were performed using longitudinal *gag* p24 sequence data from six HIV-1 infected subjects (P1-P6) carrying the HLA-B*5701 allele [5]. The subjects had different risk of progression toward AIDS based on CD4^+^ T cell count at baseline 10–11 weeks post infection (wpi) [4]. Three of these subjects (P1-P3) were classified as high-risk progressors (HRPs) and three (P4-P6) as low-risk progressors (LRPs) [5]. The average baseline viral load (VL) was 4,250 copies/mL for HRPs and 4,229 copies/mL for LRPs, while average baseline CD4^+^ T cell count was 458 cells/mm^3^ for HRPs and 1,129 cells/mm^3^ for LRPs.

**Figure 1:**
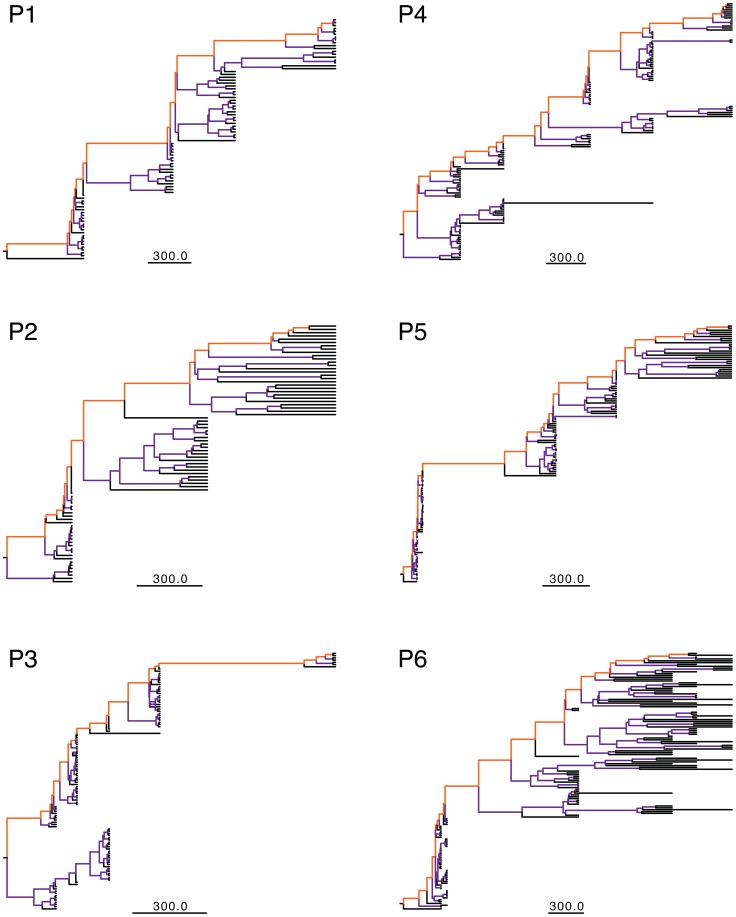
HIV-1 *gag* p24 genealogies for all six HLA-B*5701 subjects displaying different branch sets. Internal and external branches are shown for each subject (P1-P6), colored in purple and black, respectively. Orange branches indicate one of the possible backbone paths in the genealogy (see Methods). Specific estimates were based on 200 randomly sampled trees from the posterior distribution obtained with a Bayesian coalescent framework enforcing a relaxed molecular clock. Branch lengths are drawn proportional to the time scale (in days) at the bottom of each tree.

**Figure 2:**
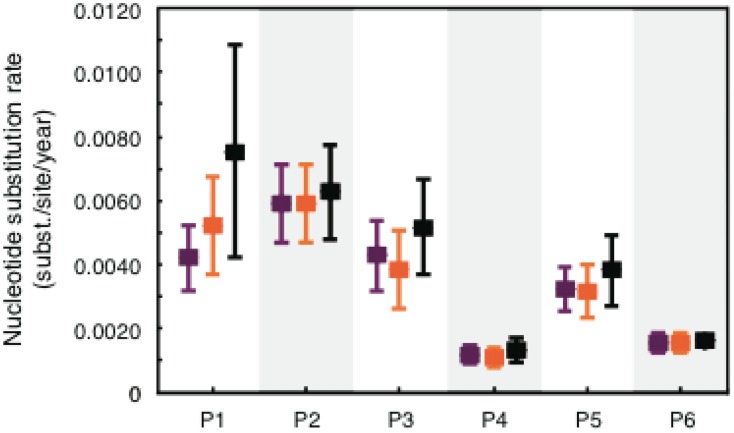
HIV-1 mean nucleotide substitution rate for different branches for all six HLA-B*5701 subjects. Estimates along internal branches (purple), backbone paths (orange) and external branches (black) are shown for the HRPs (P1-P3) and the LRPs (P4-P6).

The presence of molecular clock signal in each data set was first investigated by regression between root-to-tip divergence and sampling date on ML trees, which showed high correlation (r^2^>0.6) for each data set. HIV-1 evolutionary rate, estimated by molecular clock analysis of longitudinally sampled viral sequences, has been shown to be lower in LRPs than in HRPs [5]. However, rate estimates can be biased due to potential differences in internal and external branches of the phylogenetic tree. HIV-1 high mutation rate is expected to lead to a considerable number of deleterious mutations in the viral population, such that the most recent mutations segregating on external branches of HIV-1 phylogenies are likely to be transient [20], [28], [29], [30]. Deleterious mutations are rapidly purified and their inclusion can bias nucleotide divergence and evolutionary rate estimates, while mutation along internal branches are usually fixed. Internal and external branches were, thus, defined for 200 trees randomly sampled from the posterior distribution of HIV-1 *gag* p24 genealogies inferred with a Bayesian framework under a relaxed molecular clock (Figure 1), and mean evolutionary rates were estimated separately for each branch subset (Figure 2). In longitudinally sampled genealogies it is also possible to define the subset of branches connecting the root node with the most recent common ancestor of the sequences sampled at the last time point, which represent the surviving viral population successfully propagating over time through sequential bottlenecks driven by either positive selection or neutral genetic drift [28]. However, in HIV-1 intra-host genealogies, the last sampled sequences may not be monophyletic and different sets of backbone branches can be defined by a simple rotation around an internal branch. Therefore, a weighted average of the evolutionary rate was also calculated for the rates estimated along all the possible backbone paths of a genealogy (one example of such paths is shown by the branches highlighted in orange in Figure 1). For most patients, evolutionary rates in internal branches and backbone paths of the viral genealogies were very similar, while rates for external branches were higher (for all patients except P6) due, as expected, to an increased amount of deleterious mutations. However, the difference in mean substitution rates between HRPs and LRPs was still significant (*p*<0.05) in each analysis.

### Difference in HIV-1 nonsynonymous and synonymous rate patterns

**Figure 3:**
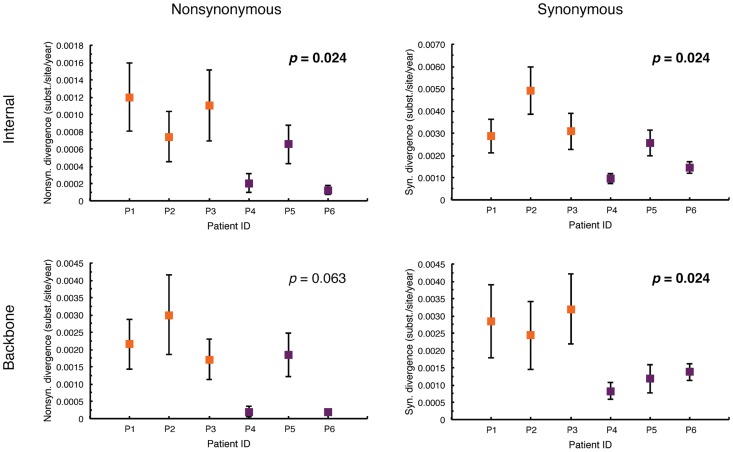
HIV-1 mean nonsynonymous and synonymous substitution rates for internal branches in HLA-B*5701 subjects. Subject- specific estimates were based on 200 randomly sampled trees from the posterior distribution obtained with a Bayesian coalescent framework enforcing a relaxed molecular clock. HRPs (P1-P3) and LRPs (P4-P6) are shown in orange and purple, respectively. The *p*-value is marked in bold when there is a significant difference between the two groups of patients. Along the y-axis, internal refers to all internal branches; backbone refers to rates averaged along all the possible backbone paths.

Gag p24 evolutionary rate differences between HRPs and LRPs were investigated in more detail by disentangling nonsynonymous (dN) and synonymous (dS) rates. Absolute dN and dS rates for all, internal, and external branches, as well as average rates for the backbone paths of the viral genealogies were estimated for each patient. The virus infecting HRPs displayed significantly higher dN rates along internal branches (Mann-Whitney U-test, *p* = 0.024) compared to the LRPs (Figure 3). There was also a trend toward higher dN rates in HRPs compared to LRPs when backbone paths (Figure 3), external or all branches (Figure S1) of the viral genealogies were analyzed. HIV-1 dS rates were significantly higher in HRPs than LRPs (Figure 3) for both internal branches (*p* = 0.024) and backbone paths (*p* = 0.024). A significant difference (*p* = 0.024) between the two groups of patients was also observed when external or all branches were analyzed (Figure S1).

**Figure 4:**
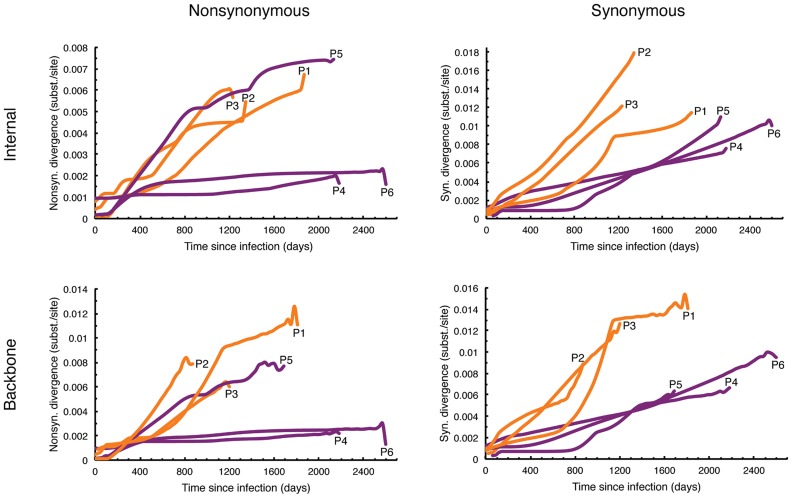
HIV-1 mean nonsynonymous and synonymous divergence for internal branches over time in HLA-B*5701 subjects. Subject- specific estimates were based on 200 randomly sampled trees from the posterior distribution obtained with a Bayesian coalescent framework enforcing a relaxed molecular clock. HRPs (P1-P3) and LRPs (P4-P6) are shown in orange and purple, respectively. Along the y-axis, internal refers to all internal branches; backbone refers to rates averaged along all the possible backbone paths.

Similarly, plots of HIV-1 dN and dS divergence over time within each patient were estimated for all and internal branches and along backbone paths. For internal branches, as well as along backbone paths, the virus infecting HRPs displayed a faster accumulation of dN substitutions over time compared to the LRPs for all patients except P5 (Figure 4). Analogous results were observed for all branches (Figure S2). There was a clear separation in dS divergence over time between the two groups of patients. The viruses infecting HRPs showed, overall, a higher number of dS substitutions over time for both internal branches and backbone paths compared to LRPs (Figure 4). Interestingly, during the first year (up to 400 days) of the infection, the accumulation of dS substitutions seemed to happen at the same rate between the two groups of patients. After the first year, the two groups began to diverge, and the virus populations in LRPs appeared to accumulate dS substitutions more slowly than those in HRPs.

### Substitution rate heterogeneity across sites and selection pressure

The observed difference in dN and dS substitution patterns between the two groups of patients could be due to strong site-to-site rate variation, which has the potential to bias the estimates [31]. To investigate this possibility within the p24 gene we estimated the coefficient of variation (CoV) of substitution rates across dS and dN sites. As expected, the analysis revealed significant across-site variation in viral dS for all data sets, although the values were lower compared to the ones estimated for the HIV-1 *env* data set analyzed in Lemey *et al*. [20]. For both dN and dS, CoVs were similar among all six patients and there was no significant difference between the HRPs and LRPs (Table 1). Therefore, it is likely that the presence of significant rate heterogeneity across dS and dN sites equally affected HIV-1 evolutionary rate estimates in both groups of patients and does not account for the observed differences.

**Table 1:** Analysis of among-site dN and dS rate variation in six HLA-B*5701 subjects.

Classification^1^	Subject	logL NS^2^	logL Dual^3^	*p* 4	CoV (*dS*)^5^	CoV (*dN*)^6^
	P1	−1524.4	−1514.3	0.00004	1.8	2.7
HRPs	P2	−1671.8	−1625.1	<0.00001	1.8	3.3
	P3	−1156.9	−1155.6	0.26	1.4	2.4
	P4	−1332.8	−1330.3	0.08	1.2	3.4
LRPs	P5	−1419.3	−1399.9	<0.00001	2.0	2.6
	P6	−1787.4	−1763.7	<0.00001	1.4	4.9

1 Classification of subjects based on baseline CD4^+^ T cell counts: HRP  =  high-risk progressors (baseline CD4 counts <750 cells/mm^3^), LRP  =  low-risk progressors (baseline CD4 counts >750 cells/mm^3^).

2 Log likelihood of the dN variable rates model.

3 Log likelihood of the dual (dS and dN) variable rates model.

4 *P*-values according to a likelihood ratio test comparing dN and dual variable rates models.

5 Coefficient of variation (standard deviation/mean) of dS rates among sites.

6 Coefficient of variation of dN rates.

Differences in mean dS between HRPs and LRPs could also be the result of different levels of purifying selection. For each branch set (all, internal, external and backbone paths) of the viral genealogies, dN/dS ratios were calculated and compared. In general, correlation of dN versus dS rates was weak (0.17–0.39), and slopes were <1, indicating signal for purifying selection rather than neutral genetic drift or positive selection (Table S1). No significant difference was observed between LRPs and HRPs (Table S2) indicating that differential purifying selection was also an unlikely explanation for the observed substitution patterns.

### Viral replication capacity and immune activation in HLA-B*5701 subjects

Since dS substitutions are neutral or nearly neutral [32], HIV-1 dS is expected to be proportional to the virus replication rate [20]. The higher dS in HRPs may be the consequence of an infection with fitter viral variants characterized by faster replication. We examined viral replication capacity (RC) for all six HLA-B*5701 subjects (1–4 time points) by using a Phenosense Gag-Pro assay (see Methods). As expected, a trend toward increased RC over time was observed in all patients likely linked to the progressive fixation of fitter variants driven by ongoing selection [33]–[35]. However, no differences in RC measurements (10–332 wpi) were apparent between LRPs and HRPs (Table 2), suggesting that the observed difference in dS may not be related to fitter (high replicative) variants infecting the HRPs.

**Table 2:** Replication capacity in six HLA-B*5701 subjects.

Classification	Subject	Weeks post infection	Replication capacity (%)^1^
		13	6.1
	P1	104	1.4
		168	9.8
HRPs		271	25
		18	5.3
	P2	109	4.7
		195	7.3
	P3	45	18.5
		60	0.1
	P4	157	4.3
		227	6.9
		316	7.1
LRPs		19	53.0
	P5	201	16.5
		309	62.5
		10	1.8
	P6	183	2.8
		332	25.0

1 Replication capacity (RC) was determined by using PhenoSense Gag-Pro assay, and expressed as a percentage of viral infectivity (luciferase activity) relative to NL4-3 reference control.

Finally, CD38 expression on CD4^+^ and CD8^+^ T cells was measured at baseline (13–17 wpi). Two HRPs (P1 and P2) displayed the highest values of CD38 expression on CD4^+^ T cells. Differences in CD38 expression on CD4^+^ or CD8^+^ T cells between the two groups of patients were not statistically significant (Table S3). Therefore, T cell activation during early infection is also an unlikely explanation for the observed difference in dS between HRPs and LRPs.

### Inverse correlation between baseline CD4^+^ T cell counts and synonymous substitution rates

**Figure 5:**
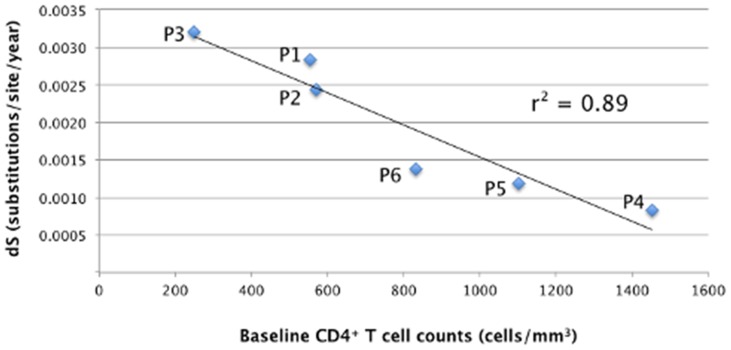
HIV-1 mean synonymous substitution rates along backbone paths *vs*. CD4^+^ T cell count at baseline for each subject. HIV-1 synonymous rates (dS) in *gag* p24 within each HLA-B*5701 subject (y-axis) were plotted against baseline CD4^+^ T cell counts (10–11 wpi). The squared correlation coefficient (r^2^) is given in the graph and resulted highly significant (*p* = 0.002).

In order to identify other potential mechanisms behind the observed differences in viral evolutionary rate, correlation between mean dN or dS for different branch sets (all, internal, external and backbone paths) and clinical parameters for each patient (baseline CD4 count, baseline VL, CD4 slope, VL slope and baseline T cell activation) were investigated (Table S4). The only strong correlation found (r^2^ = 0.9) was between weighted average of dS estimated along possible backbone paths and baseline CD4^+^ T cell counts (Figure 5). In particular, higher baseline CD4^+^ T cell counts (10–11 wpi) were correlated with lower dS, indicative of lower replication rates and longer viral generation times in the LRPs compared to HRPs. The inverse correlation was highly significant (*p* = 0.002), even after Bonferroni correction (*p* = 0.09).

## Discussion

The present work investigated in depth the relationship between dS and dN viral evolutionary rate and risk of disease progression in HIV-1-infected subjects carrying the HLA-B*5701 allele. A recent study carried out on the same cohort has shown that HRPs have significantly lower polyfunctional CD8+ T cell responses, as well as higher viral evolutionary rate than LRPs [5]. The study also noticed that dS and dN changes, calculated by pairwise comparisons, were higher in HRPs than LRPs. However, the exact mechanism driving HIV-1 faster evolutionary rate in HRPs and its clinical implications remained obscure. Herein, absolute rates of dS and dN substitutions were estimated by Bayesian molecular clock analysis from longitudinally sampled HIV-1 *gag* p24 sequences along different branches of the viral genealogies. The evolutionary analyses and the comparison of dN and dS with various clinical, immunological and virological parameters resulted in three major and novel findings, which provide a potential mechanism for the initial observations reported in Norstrom *et al*. (2012). First, it was shown that the virus infecting LRPs exhibited significantly lower dN and dS divergence over time compared to HRPs. Second, no significant difference in site-to-site variation of dS or in dN/dS ratios along different branches of the HIV-1 genealogies was observed between the two groups of patients. This indicates that differences in rate heterogeneity across synonymous sites or purifying selection were unlikely to be the cause of lower viral dS in LRPs. Third, the analysis detected a strong inverse correlation between HIV-1 dS, which is directly proportional to the virus replication rate [33], [36], and baseline (10–11 wpi) CD4^+^ T cell count.

Changes in absolute HIV-1 dN and dS rates have been investigated previously [34], by analyzing *env* sequences from the Shankarappa *et al*. (1999) data set – nine HIV-1 infected patients followed longitudinally from the time of seroconversion [34]. However, seven out of nine patients in that data set received antiretroviral treatment during the follow-up time, their HLA-type was unknown and no data on *in vitro* viral RC or immune activation were available, making it difficult to disentangle the different factors that may have contributed to the interplay between viral evolution and disease progression. On the other hand, the present study examined untreated HLA-B*5701 subjects with different risk of disease progression, where HIV-1 evolutionary patterns could be compared to *in vitro* viral RC data, using a novel Gag-pro phenotypic assay, as well as immune activation data and a number of clinically relevant parameters.

Using the Shankarappa data set [34], Lemey *et al*. (2007) provided some evidence that slow HIV-1 disease progression can be predicted by lower dS rates, which are indicative of the underlying viral replication dynamics. In agreement with our finding, they did not detect significant differences in rate heterogeneity across synonymous sites between patients with different rates of disease progression. Rate heterogeneity, however, was generally higher than the one estimated for the viruses infecting the subjects enrolled in the present study, which likely reflects the higher diversity in *env* gp120 compared to *gag* p24 region. Lemey *et al*. (2007) also suggested that the slower replication dynamics of HIV-1 in patients with slow disease progression could depend on the state of immune activation of the host. Indeed, T cell activation (defined as the expression of CD38 on the T cells) is one of the strong predictors of progression to AIDS [22], [23], [25], [37], [38]. Nevertheless, within the HLA-B*5701 subjects studied herein no significant differences in T cell activation was observed. In addition, our analysis showed no differences in viral RC between the HRPs and LRPs. A trend toward increasing RC over time was observed in viruses sampled from patients with RC longitudinal data available, which is expected as a result of the continuous emergence and fixation of fitter viral variants over time [33]. HRPs displayed significantly higher dN rate along internal branches of the viral genealogies compared to the LRPs, which may be indicative of a higher rate of adaptation in the HRPs. It is important to notice, however, that the small sample size of our cohort requires a certain caution before drawing firm conclusions and no samples from earlier than 11 wpi were available to compare whether RC differed between HRPs and LRPs during primary infection. Moreover, the RC assay tested only one part of the viral genome that may not fully capture viral replication capacity. Yet, the data suggest that neither T cell activation nor an initial infection with fitter viral variants would explain the difference in dS substitution patterns between HRPs or LRPs carrying the HLA-B*5701 allele.

An intriguing alternative can be hypothesized by considering the highly significant inverse correlation between baseline CD4^+^ T cell count and average dS rates along branches representing lineages effectively propagating through time. The finding suggests a mechanistic link between CD4^+^ T cell count and the virus replication rate [33], [36], by indicating that HLA-B*5701 subjects with CD4^+^ T cell counts >750 cells/mm^3^ within the first 10–11 weeks of the infection will keep HIV-1 replication under better control during the subsequent years. This observation is in agreement with earlier results showing that subjects with a stronger immune system during early infection exhibit more constrained viral evolution, probably linked to a more robust HLA-B*5701-specific CD8^+^ T cell response [5]. In other words, the higher polyfunctional responses observed in these subjects [5] coupled with a larger number of CD4^+^ T cells during early infection may ultimately result in an overall slower *in vivo* replication rate of the virus. There is evidence that emergence of escape mutations in p24, as a consequence of CD8^+^ T cell responses, can negatively affect viral fitness [16], and thereby be indirectly responsible for control of viral replication, longer generation times, and lower risk to progress to AIDS.

Replication rates can also depend on the ability of the viral strains to replicate in different environments [16]. Differences in the contribution of latent HIV-1 reservoirs, such as resting memory CD4^+^ T cells, to the circulating virus population can impact mean generation times and replication rates even though the may produce only a fraction of circulating viruses [26], [27]. Further work will be necessary to clarify the relationship between HIV-1 generation times and replication dynamics in different viral reservoirs. Even though we included more HLA-B*5701 patients than in previous studies, our sample size remains small and conclusions need to be taken with caution. Regardless, our findings provide, for the first time, a possible evolutionary mechanism for different risk of disease progression in HLA-B*5701 subjects. They indicate that subjects who maintain high CD4^+^ T cell counts in early infection are more likely to control HIV-1 replication for an extended time and that synonymous substitution rates, which are proportional to *in vivo* replication rates, could be used as a novel evolutionary marker of disease progression.

## Materials and Methods

### Ethics statement

The University of California, San Francisco (UCSF) Committee on Human Research, the Regional Ethical Council in Stockholm, Sweden (2008/1099-31), and the University of Florida review board approved this study. All patients provided written informed consent and all clinical investigations were conducted according to the principles expressed in the Declaration of Helsinki.

### Patients' characteristics

The study included six untreated HIV-1 subtype B infected men (P1-P6), all carrying the HLA allele B*5701, from the OPTIONS cohort [39]. Patients were enrolled within six months of HIV seroconversion and followed longitudinally. Patients' details have been described in a previous study [5]. Briefly, five of them were men who have sex with men (MSM) and one (P5) was an injecting drug user (IDU).

### Estimating absolute rates of nonsynonymous and synonymous substitutions

Each patient-specific data set included HIV-1 *gag* p24 sequences obtained by single genome sequencing of longitudinal plasma samples as previously described [5], [40]. GenBank accession numbers for the sequences analyzed in this study are: JX234575-JX235332. All sequences included in the present study were non-recombinant, as previously described [5]. The presence of molecular clock signal in each patient data set was investigated by regression between root-to-tip divergence and sampling date using ML likelihood trees inferred with the best fitting nucleotide substitution model, chosen by a hierarchical likelihood ratio test, as previously described [5]. For each data set, the Markov chain Monte Carlo (MCMC) sampler implemented in BEAST 1.7 [41], was used to obtain a posterior distribution of trees under a relaxed molecular clock model with the best fitting population prior (according to estimated Bayes Factors) [5], [42]. The selected population prior for each data set was the Bayesian skyline plot. The approach to infer synonymous and nonsynonymous substitution rates, and to explore how these rates change through time, is an empirical extension of the coalescent-based Bayesian relaxed clock models [20], [42]. Briefly, a subsample of 200 trees was randomly selected from posterior distribution and used to re-estimate branch lengths proportional to either nonsynonymous or synonymous substitutions according to the method described in Lemey *et al* [20]. For each clock-like genealogy, the rate of absolute nonsynonymous and synonymous substitutions was estimated including all branches in the genealogy, as well as internal and external branches only. The weighted average of the evolutionary rate was also calculated for the rates estimated along all the possible backbone paths of a genealogy (weighted by the number of branches along each path). Backbone paths represent lineages propagating (i.e. effectively surviving) from root node to sequences sampled at the last time point through sequential population bottlenecks. For each data set, HIV-1 among-site nonsynonymous and synonymous rate variation was analyzed by comparing two nested models with the likelihood ratio test: the constant rate variation model, which assumes that neither nonsynonymous nor synonymous rates vary across sites, and the *dual* random effects likelihood (REL) model, where site-specific nonsynonymous or synonymous rates are drawn from independent general discrete distributions with three rate categories [31], [43]. The dual REL model estimates the coefficient of variation (CoV), defined as standard deviation/mean for nonsynonymous or synonymous rates across sites. A large CoV and a low *p*-value for the test comparing the dual model with the null hypothesis (constant model) that CoV  = 0 indicate significant rate variation from codon to codon in the alignment.

### Gag-pro mediated replication capacity and immune activation data

Viral replication capacity (RC) was measured in *vitro* using the PhenoSense Gag-Pro assay [44]. Sequences of *gag* and protease genes were amplified from patients' plasma by RT-PCR and transferred into a resistance test vector (RTV) containing a luciferase reporter gene. Transfections of HEK293 cells with patient-derived *gag-pro* RTVs and an amphotropic murine leukemia virus envelope expression vector were performed to generate pseudovirus stocks for infection of HEK293 cells. Gag-pro mediated RC was determined by measuring the viral infectivity (luciferse activity) of patient-derived pseudoviruses relative to NL3-4, the reference control, and expressed as a percentage. Immune activation data were also available for all subjects. The proportion of CD4^+^ and CD8^+^ T cells expressing CD38 was measured at 13–17 weeks post infection (wpi), as previously described [23].

### Statistical analysis

A one-tail Mann-Whitney U-test was carried out with an online calculator using the normal approximation (http://elegans.som.vcu.edu/leon/stats/utest.html) to assess whether substitution rates in HRPs were significantly higher than in LRPs. Slopes of CD4^+^ T cell counts and viral load (VL) were obtained from least squares regression of log-transformed CD4 counts and VL over time (years). Model coefficients were back transformed and converted from proportions to percentage effect by subtracting one and multiplying by 100 to obtain individual estimates of percent change over time. Mean nonsynonymous and synonymous rates for different set of branches (all, internal, external and backbone paths) were compared to the corresponding clinical parameters of each patient (baseline CD4 count, baseline VL, CD4 slope, VL slope and baseline T cell activation) using Pearson's linear correlation to calculate the associated t-values and assess significance. All *p*-values obtained from applying any test statistic multiple times were adjusted with the Bonferroni correction.

## Supporting Information

Figure S1 Mean nonsynonymous (dN) and synonymous (dS) rates for all and external branches in HLAB*5701 subjects. HRPs (P1-P3) and LRPs (P4-P6) are shown in orange and purple, respectively. There is a significant difference in dS divergence between the two groups of patients for all and external branches (marked in bold).
(PDF)

Figure S2 Mean nonsynonymous (dN) and synonymous (dS) divergence for all branches over the course of HIV infection in HLA-B*5701 subjects. HRPs (P1-P3) and LRPs (P4-P6) are shown in orange and purple, respectively.
(PDF)

Table S1 Linear regression slopes of nonsynonymous (dN) vs. synonymous (dS) substitution rates estimated for all HLA-B*5701 subjects.
(PDF)

Table S2 Ratio of mean substitution rates (dN/dS) for each HLA-B*5701 subject.
(PDF)

Table S3 T cell activation in six HLA-B*5701 subjects.
(PDF)

Table S4 Linear regression correlation coefficients of clinical markers vs. substitution rate estimates for all HLA-B*5701 subjects.
(PDF)
