# Correction: Baseline CD4+ T Cell Counts Correlates with HIV-1 Synonymous Rate in HLA-B*5701 Subjects with Different Risk of Disease Progression

**DOI:** 10.1371/journal.pcbi.1004000

**Published:** 2014-11-03

**Authors:** The PLOS Computational Biology Staff

There is an error in the author list of this manuscript. The eighth author's name is spelled incorrectly. The correct name is: Annika C. Karlsson.
