# Supplementary material for: Baseline CD4+ T Cell Counts Correlates with HIV-1 Synonymous Rate in HLA-B*5701 Subjects with Different Risk of Disease Progression
Source: PLoS Comput Biol. 2014 Sep 4;10(9):e1003830. doi: 10.1371/journal.pcbi.1003830 (PMC4154639; doi:10.1371/journal.pcbi.1003830)
Supplement: Figure S1 — Mean nonsynonymous (dN) and synonymous (dS) rates for all and external branches in HLAB*5701 subjects. HRPs (P1-P3) and LRPs (P4-P6) are shown in orange and purple, respectively. There is a significant difference in dS divergence between the two groups of patients for all and external branches (marked in bold). (PDF) [file pcbi.1003830.s001.pdf]

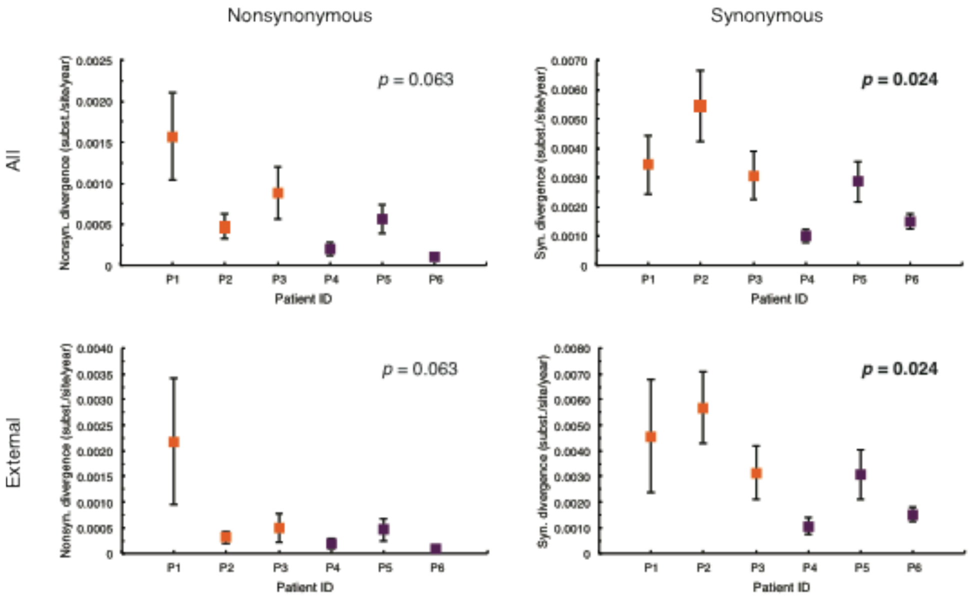

Figure S1. Mean nonsynonymous (dN) and synonymous (dS) rates for all and external branches in HLAB*5701 subjects. HRPs (P1-P3) and LRPs (P4-P6) are shown in orange and purple, respectively. There is a significant difference in dS divergence between the two groups of patients for all and external branches (marked in bold).
