# Supplementary material for: Baseline CD4+ T Cell Counts Correlates with HIV-1 Synonymous Rate in HLA-B*5701 Subjects with Different Risk of Disease Progression
Source: PLoS Comput Biol. 2014 Sep 4;10(9):e1003830. doi: 10.1371/journal.pcbi.1003830 (PMC4154639; doi:10.1371/journal.pcbi.1003830)
Supplement: Table S1 — Linear regression slopes of nonsynonymous (dN) vs. synonymous (dS) substitution rates estimated for all HLA-B*5701 subjects. (PDF) [file pcbi.1003830.s003.pdf]

SUPPORTING INFORMATION FILE 1

Table S1. Linear regression slopes of nonsynonymous (dN) *vs*. synonymous (dS) substitution rates estimated for all HLA-B*5701 subjects.

	All[1]	Internal[2]	Backbone Paths[3]	External[4]
**Slope**	0.14	0.20	0.78	0.21
**R^2**	0.17	0.38	0.39	0.21
***p*-value**	0.21	0.10	0.09	0.18

[1] HIV-1 dN and dS were estimated for each data set by including all branches of the viral genealogy.
[2] dN and dS estimates for internal branches only.
[3] Average dN and dS estimated along each possible backbone path.
[4] dN and dS rate estimates for external branches only.
