# Supplementary material for: Baseline CD4+ T Cell Counts Correlates with HIV-1 Synonymous Rate in HLA-B*5701 Subjects with Different Risk of Disease Progression
Source: PLoS Comput Biol. 2014 Sep 4;10(9):e1003830. doi: 10.1371/journal.pcbi.1003830 (PMC4154639; doi:10.1371/journal.pcbi.1003830)
Supplement: Table S2 — Ratio of mean substitution rates (dN/dS) for each HLA-B*5701 subject. (PDF) [file pcbi.1003830.s004.pdf]

## SUPPORTING INFORMATION FILE 2

**Table S2. Ratio of mean substitution rates (dN/dS) for each HLA-B*5701 subject.**

| Classification | Subject | All[1] | Internal[2] | Backbone Paths[3] | External[4] |
|---|---|---|---|---|---|
| HRPs | P1 | 0.46 | 0.42 | 0.76 | 0.48 |
| | P2 | 0.09 | 0.15 | 1.37 | 0.05 |
| | P3 | 0.29 | 0.36 | 0.54 | 0.16 |
| LRPs | P4 | 0.2 | 0.21 | 0.24 | 0.18 |
| | P5 | 0.2 | 0.26 | 1.56 | 0.15 |
| | P6 | 0.07 | 0.09 | 0.13 | 0.07 |

[1] HIV-1 dN and dS substitution rates were estimated for each data set by including all branches of the viral genealogy.
[2] dN and dS rate estimates for internal branches only.
[3] Average dN and dS estimated along each possible backbone path.
[4] dN and dS rate estimates for external branches only.
