# Supplementary material for: Baseline CD4+ T Cell Counts Correlates with HIV-1 Synonymous Rate in HLA-B*5701 Subjects with Different Risk of Disease Progression
Source: PLoS Comput Biol. 2014 Sep 4;10(9):e1003830. doi: 10.1371/journal.pcbi.1003830 (PMC4154639; doi:10.1371/journal.pcbi.1003830)
Supplement: Table S3 — T cell activation in six HLA-B*5701 subjects. (PDF) [file pcbi.1003830.s005.pdf]

## SUPPORTING INFORMATION FILE 3

**Table S3. T cell activation in six HLA-B*5701 subjects.**

| Classification | Subject | Weeks post infection | $CD38^+CD4^+$ T cells (%)[1] | $CD38^+CD8^+$ T cells (%)[2] |
|---|---|---|---|---|
| HRPs | P1 | 13 | 30.2 | 5.6 |
| | P2 | 13 | 47.2 | 57.1 |
| | P3 | 17 | 4.9 | 13.5 |
| LRPs | P4 | 13 | 16.9 | 1.0 |
| | P5 | 13 | 3.3 | 6.2 |
| | P6 | 13 | 22.9 | 13.5 |
| *P*-value[3] | | | 0.3 | 0.4 |

[1] Percentage of $CD4^+$ T cells expressing CD38.
[2] Percentage of $CD8^+$ T cells expressing CD38.
[3] A two-tailed Mann Whitney U test was used to test if there was any significant difference between HRPs and LRPs.
