# Supplementary material for: Baseline CD4+ T Cell Counts Correlates with HIV-1 Synonymous Rate in HLA-B*5701 Subjects with Different Risk of Disease Progression
Source: PLoS Comput Biol. 2014 Sep 4;10(9):e1003830. doi: 10.1371/journal.pcbi.1003830 (PMC4154639; doi:10.1371/journal.pcbi.1003830)
Supplement: Table S4 — Linear regression correlation coefficients of clinical markers vs. substitution rate estimates for all HLA-B*5701 subjects. (PDF) [file pcbi.1003830.s006.pdf]

Table S4. Linear regression correlation coefficients (r)[1] of clinical markers[2] vs. substitution rate estimates[3] for all HLA-B*5701 subjects.

Clinical markers	dN_rate_all	dN_rate_int	dN_rate_bb	dN_rate_ext	dS_rate_all	dS_rate_int	dS_rate_bb	dS_rate_ext
**CD4**	-0.50 (0.16) [1]	-0.74 (0.046) [1]	-0.47 (0.17) [1]	-0.35 (0.25) [1]	-0.60 (0.11) [1]	-0.66 (0.08) [1]	-0.89 (0.002) [0.08]	-0.57 (0.12) [1]
**VL**	0.10 (0.43) [1]	-0.17 (0.37) [1]	-0.0007 (0.5) [1]	0.26 (0.31) [1]	0.06 (0.46) [1]	-0.01 (0.49) [1]	0.006 (0.50) [1]	0.14 (0.39) [1]
**CD4 (slope)**	-0.61 (0.1) [1]	0.53 (0.14) [1]	-0.76 (0.04) [1]	-0.63 (0.09) [1]	-0.74 (0.05) [1]	0.03 (0.48) [1]	-0.40 (0.22) [1]	-0.85 (0.017) [0.59]
**VL (slope)**	0.22 (0.33) [1]	-0.48 (0.17) [1]	-0.11 (0.42) [1]	0.04 (0.47) [1]	-0.08 (0.44) [1]	-0.63 (0.09) [1]	0.52 (0.14) [1]	-0.12 (0.41) [1]
**$CD38^+CD4^+$**	0.11 (0.42) [1]	0.02 (0.48) [1]	0.54 (0.13) [1]	0.18 (0.37) [1]	0.60 (0.10) [1]	0.53 (0.14) [1]	0.17 (0.38) [1]	0.61 (0.10) [1]
**$CD38^+CD8^+$**	-0.15 (0.39) [1]	0.10 (0.42) [1]	0.70 (0.06) [1]	-0.25 (0.32) [1]	0.81 (0.027) [0.97]	0.83 (0.02) [0.98]	0.29 (0.29) [1]	0.65 (0.08) [1]

[1] *P*-values for the linear correlation are given in parentheses. *P*-values after Bonferroni correction are given within square brackets. Statistically significant values after Bonferroni are highlighted in red.
[2] Clinical markers (first column from the left) include: CD4 = $CD4^+$ T cell counts at baseline (10-11 wpi); $CD38^+CD4^+$ = Percentage of $CD4^+$ T cells expressing CD38 at baseline (13-17 wpi); VL = viral load at baseline (10-11 wpi); $CD38^+CD8^+$ = Percentage of $CD8^+$ T cells expressing CD38 at baseline (13-17 wpi); VL (slope) = % VL change per year; CD4 (slope) = % CD4 counts change per year.
[3] Substitution rate estimates include: dN_rate_all = mean nonsynonymous rate estimated for each data set by including all branches in the HIV-1 genealogy; dN_rate_int = mean nonsynonymous rate estimate for internal branches only; dN_rate_bb = average nonsynonymous rate along each backbone path; dN_rate_ext = mean nonsynonymous rate estimate for external branches only; dS_rate_all = mean synonymous rate estimate for all branches; dS_rate_int = mean synonymous rate estimate for internal branches only; dS_rate_bb = average synonymous rate along each backbone path; dS_rate_ext = mean synonymous rate estimate for external branches only.
